# Finding My Way-Advanced: can a web-based psychosocial intervention improve the mental quality of life for women with metastatic breast cancer vs attention-control? Study protocol of a randomised controlled trial

**DOI:** 10.1186/s12885-022-10410-z

**Published:** 2022-12-24

**Authors:** Lisa Beatty, Emma Kemp, Phyllis Butow, Afaf Girgis, Nicholas Hulbert-Williams, Billingsley Kaambwa, Penelope Schofield, Jane Turner, Richard Woodman, Frances Boyle, Anthony Daly, Amanda Jones, Belinda Kiely, Nicholas Zdenkowski, The FMW-A Authorship Group, Bogda Koczwara

**Affiliations:** 1 Flinders University, Adelaide, Australia; 2 University of Sydney, Sydney, Australia; 3 University of NSW, Sydney, Australia; 4 Edge Hill University, Ormskirk, UK; 5 Swinburne University, Melbourne, Australia; 6 Peter MacCallum Cancer Centre, Melbourne, Australia; 7 University of Queensland, St Lucia, Australia; 8 Mater, Sydney, Australia; 9 Cancer Council South Australia, Adelaide, Australia; 10 Southern Adelaide Local Health Network, Bedford Park, Australia; 11 Macarthur Cancer Therapy Centre, Campbelltown Hospital, Campbelltown, Australia; 12 University of Newcastle, Newcastle, Australia

**Keywords:** Metastatic breast cancer, Psycho-oncology, Digital health, Intervention, QOL, Distress, CBT, RCT

## Abstract

**Background:**

Women living with metastatic breast cancer (MBC) are at risk of significantly impaired quality of life (QOL), symptom burden, distress and fear of progression, and unmet needs, yet they face barriers to accessing evidence-based psychosocial treatments. Our group therefore developed *Finding My Way-Advanced (FMW-A),* a web-based self-guided psychosocial program for women with MBC. This study aims to assess its efficacy in improving mental and other QOL domains, distress, fear of progression, unmet needs, and health service utilisation.

**Methods:**

The multi-site randomised controlled trial (RCT) will enrol 370 Australian participants. Eligible participants are adult (18 years +) women diagnosed with MBC, with a life expectancy of 6 months or more, with sufficient English-language literacy to provide informed consent. Participants will be identified, screened and referred from one of 10 Australian sites, or via self-referral in response to advertisements. Participants complete four online questionnaires: prior to accessing their program (‘baseline’), 6 weeks later (‘post-intervention’), then 3 months and 6 months post-intervention. Consenting participants will be randomised to either *FMW-A* (intervention), or Breast Cancer Network Australia’s (BCNA) online/app resource *My Journey* (minimal intervention attention-control). This is a single-blind study, with randomisation computer-generated and stratified by site. *FMW-A* is a 6-module program addressing some of the most common issues experienced by women with MBC*,* with BCNA control resources integrated within the ‘resources’ section*.* All modules are immediately accessible, with an additional booster module released 10 weeks later. The primary outcome is mental QOL; statistical criteria for superiority is defined as a 4-point difference between groups at post-treatment. Secondary outcomes include other QOL domains, distress, fear of progression, health service use, intervention adherence, and user satisfaction.

**Discussion:**

This will be the first adequately powered RCT of a self-directed online intervention for women with MBC. If efficacious, *FMW-A* will help address two national key priorities for management of MBC – enhancing QOL and reducing symptom burden. *FMW-A* has the potential to address unmet needs and overcome access barriers for this overlooked population, while reducing health system burden.

**Trial registration:**

The study was registered prospectively with the ANZCTR on 29/10/2021. Trial ID ACTRN12621001482853p. https://anzctr.org.au/Trial/Registration/TrialReview.aspx?id=382714&isReview=true

**Supplementary Information:**

The online version contains supplementary material available at 10.1186/s12885-022-10410-z.

## Background

In 2020 alone, 2.3 million women globally were diagnosed with breast cancer, and 7.8 million women alive were diagnosed with breast cancer in the past 5 years, making it the world’s most prevalent cancer [1]. Of those diagnosed, approximately 5% would have received the diagnosis of metastatic breast cancer (MBC) as their first diagnosis (‘de novo’), and a further 10% experienced a recurrence/progression of prior primary breast cancer [2]. Despite improved treatments and survival duration, with some women experiencing extended survival of 10 years or longer [3], MBC remains the leading cause of female cancer mortality, with an estimated 685,000 deaths globally in 2020 [1].

Women receiving a diagnosis of MBC experience significant challenges; in addition to managing their awareness of premature mortality, they also face a more proximal future of progressing illness; physical symptoms, including pain, fatigue, and insomnia; and resulting functional limitations, such as reduced mobility or ability to undertake usual occupation/roles [4, 5]. Clinically elevated distress, psychiatric disorders, and impaired quality of life (QOL) typically occur in 35-43% of women following diagnosis of MBC [4, 5], compared to 20% in the general community. Left untreated, distress and impaired QOL can result in increased health burden [6] via reduced ability to tolerate physical symptoms and treatment toxicities, which then require further treatment [7]; reduced adherence to cancer treatments or lifestyle recommendations [8]; and increased requests for additional investigations outside scheduled visits [9]. As a result, lead national cancer control agencies argue for managing symptoms and optimising QOL as key treatment targets for MBC [2].

Yet the high levels of unmet needs, distress, symptom burden, and impaired QOL that women with MBC experience remain largely unaddressed, with research finding these women are underserviced and receive less support than women with early-stage disease [10]. Indeed, a 2015 Breast Cancer Network Australia (BCNA) survey of 582 individuals with MBC found that 68% of respondents had unmet needs, with the top three being fear of progression, fatigue, and uncertainty about the future [11]. ‘Self-care’ (including web-based interventions) was the preferred management strategy for the latter two needs, and the second-preferred option (after oncologist-involvement) for managing fear of cancer progression.

While psychological interventions have an arguably prominent role in managing all three of these unmet needs, few resources have been developed for this population, particularly resources that utilise self-care and support self-management. Indeed, our 2018 systematic review of interventions for MBC identified only 15 RCTs that have been conducted to date [12]: Overall, interventions improved distress, coping, and pain. For quality of life, sleep, and fatigue, the evidence was insufficient to form recommendations [12]. Since 2018, four additional RCTs have been published (two group-based, one individual-based, one low intensity) [13–16]. Three were pilot trials which found evidence of feasibility [13–15], while the fully powered RCT of a 12-month weekly group program found evidence of improved depression, anxiety, and QOL compared to a no-treatment control [16]. Overall, group therapy has the strongest evidence base with nine published trials [12, 16], but had the correspondingly poorest uptake/adherence [12]. The intensity of these services/interventions precludes participation by women with high symptom burden, who live in rural/remote areas, are living with socioeconomic disadvantage, or have occupational or carer demands. Personal barriers to seeking psychological interventions also exist, including perceived stigma [17], and fear of being distressed by others’ decline or death [18]. There are also significant workforce barriers, where the demand for existing psychological services exceeds supply [19]. In contrast, while low-intensity interventions (to date) have the weakest evidence base (five trials published: three telephone-based, two expressive writing, nil online), they have the highest uptake and adherence [12, 14]. In sum, given women with MBC face intensive medical treatment schedules, are frequently unable to commit to future appointments, and may have compromised health status [11, 12], future research needs to design and trial interventions that can yield efficacy while minimising participation burden.

Digital health tools, such as web-based therapy, could address these barriers. Given digital health tools provide 24/7 access at a time and place convenient to the patient, and reduce obligations to spend valued time and money in healthcare settings, this mode of providing psychosocial care has particular pertinence to women with MBC [20]. Furthermore, digital interventions are easily scalable, can be immediately updated as new evidence emerges, and provide the flexibility to explore content in a non-linear way, with patients able to access information pertinent to their most immediate needs [21]. While there are no published online intervention RCTs in this population, women with MBC are already seeking cancer-related information on the internet [20] and are receptive to the online provision of a psychosocial program [22]. The rapidly building evidence base in *early-stage* cancer populations for improving distress [23, 24], depression and anxiety [25], fear of recurrence [26], QOL [27, 28], and health service utilisation [27], can help to inform future MBC-specific intervention development.

Our team recently developed and usability-tested an online program to support women with MBC: *Finding My Way-Advanced (FMW-A)* [29]. FMW-A was co-designed, with content initially sourced and adapted from our evidence-based *Finding My Way* web-program for people with *early-stage* cancer [27, 28]. The content was authored by a senior clinical psychologist with expertise in psycho-oncology (LB), with iterative rounds of input and feedback from a multidisciplinary co-design team including consumers, clinicians, and academics. A qualitative think-aloud protocol was then implemented to test usability of the resulting 6-module prototype, with women living with MBC accessing up to three modules with an interviewer sitting alongside. Feedback summarised the supportive and informative nature of the program, supplemented by comments about broadly relatable content. However, within themes, diverging experiences emerged regarding navigability, worksheets and layout. Participants noted that having/making time for the intervention would be important to program engagement [29]. Pragmatic issues were corrected prior to feasibility pilot testing, which demonstrated feasibility via an uptake rate of 61%, a retention rate of 66%, and an engagement level of 2.3 modules [30], which was in line with prior online intervention research, and sufficient to impact outcomes [27].

Digital health tools have the potential to facilitate scalable access to cost-effective interventions and address inequity, however none have been trialled in a large-scale RCT in MBC. While digital health solutions have proliferated in the general community, they lack the required essential evidence of efficacy, safety, acceptability, accessibility, and content-relevance for this vulnerable population [31].

This study aims to address the unmet needs of women diagnosed with MBC to improve their QOL and wellbeing, and ultimately reduce the health system burden [4–6].

## Specific objectives

### Primary objective

The primary objective of the *Finding My Way-Advanced* randomised controlled trial (RCT) is to evaluate the efficacy of the intervention (‘FMW-A’) compared with an online minimal-intervention attention control (‘control’) in improving mental QOL, as measured by the ‘emotional functioning’ subscale of the European Organisation for Research and Treatment of Cancer Quality of Life Core Questionnaire (EORTC QLQ C-30), in women with MBC. The term ‘mental QOL’ was selected for this study to enable easier comparison to the broader QOL literature where mental QOL is the predominant term.

### Secondary objectives

Secondary objectives are to:

i. Evaluate the efficacy of FMW-A compared to control in improving other health-related QOL domains, psychological distress, fear of progression, unmet needs, and health service utilisation in women with MBC.
ii. Describe uptake to the RCT and adherence to the program.
iii. Evaluate the cost-effectiveness of the program.

### Hypotheses

Primary hypothesis:

1. Intervention participants will report greater improvements in the primary outcome of mental QOL (MQOL) compared to minimal-intervention control participants from baseline to post-intervention, with effects sustained at 3- and 6-month follow-ups.

Secondary hypotheses:

1. Intervention participants will report greater improvements from baseline to post-treatment in secondary well-being outcomes, i.e., physical, social, cognitive, role and global QOL domains; fear of progression; psychological distress; and unmet needs, compared to control participants, with effects sustained at 3- and 6-month follow-ups.
2. Intervention participants will have lower health service utilisation and public hospital admissions (indicator of cost savings to the health service) over the 6-month follow-up period, compared to control participants.

## Methods and design

### Study design and setting

**Fig. 1:**
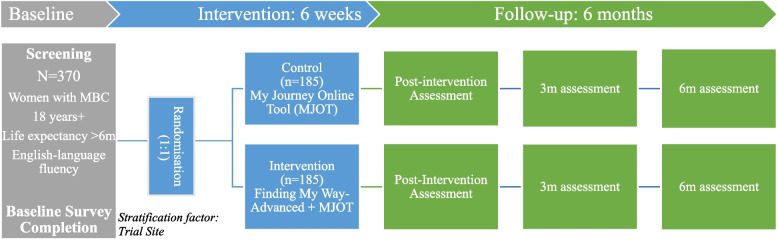
Finding My Way-Advanced Study schema

A multicentre, single-blind parallel RCT, with randomisation at the participant level and stratification by recruitment site. This trial adopts the SPIRIT guidelines for clinical protocols [32], and Fig. 1 outlines the study schema.

All RCT participation will occur via two web programs. Research survey data will be collected on Qualtrics (hosted by Flinders University) and the FMW-A web program will be hosted on ManageWP with oversight by Flinders University staff.

### Inclusion criteria

Women (1) with a diagnosis of MBC, (2) aged 18 years or older, with (3) estimated life expectancy longer than 6 months (as per treating clinician’s estimate) and (4) sufficient English for informed consent and program comprehension, will be eligible.

### Exclusion criteria

No internet access or active email address, or previous medical history of dementia or cognitive impairment. Women currently receiving face-to-face psychological services are still eligible to participate (i.e., are not excluded). Women currently enrolled in other active psychosocial intervention trials will be excluded to avoid contamination effects, while those enrolled in observational studies will remain eligible.

### Ethics approval and consent to participate

This study has received ethical approval from the Southern Adelaide Clinical Human Research Ethics Committee (SAC-HREC 2021/HRE00407). Study participants provide online written consent via selecting check-box options for each component of participation, followed by confirming overall consent by clicking the ‘Submit Consent’ button.

### Recruitment

Eligible participants will be recruited via two methods.

**Direct approach**: Women will be screened for eligibility by cancer clinicians then approached by research assistants (RAs) at the 10 participating health sites (Flinders Medical Centre, South Australia; Royal Brisbane & Women’s Hospital, Queensland; Canberra Hospital, Australian Capital Territory (ACT); National Capital Private Hospital, ACT; Peter MacCallum Cancer Centre, Victoria; Lake Macquarie Private Hospital, New South Wales (NSW); Maitland Private Hospital, NSW; Mater North Sydney Hospital, NSW; Chris O’Brien Lifehouse, NSW; Campbelltown Hospital, NSW). CIs and AIs are affiliated with each of these recruitment sites and research assistants will be employed in each state to facilitate and oversee recruitment at each recruiting hospital. Women who are screened as ineligible will receive standard care as usual.

**Self-selection:** In response to advertisements and promotion through *professional networks* (e.g., McGrath Foundation; BCNA; Cancer Council SA), *breast cancer advocacy groups / agencies* (e.g., Cancer Voices Australia), and *research databases* (e.g., Register4, BCNA Review & Survey group). Participants who self-refer to the study will self-confirm their eligibility as part of the online consent process.

### Interventions / groups

#### Intervention: Finding My Way-Advanced

FMW-A is a 6-module psychosocial program that addresses commonly experienced concerns raised by women with MBC (see Table 1). Module content is provided in a multimedia format, with video and written personal accounts from women living with MBC, educational videos from health care professionals, self-management activities, downloadable relaxation-mindfulness audio files, print psychoeducation with audio-conversion options, with illustrations and imagery throughout. Two key strategies facilitate usage. First, personalisation and free-choice access: Based on pilot feedback and adult learning literature [31], after viewing an introductory ‘how to use’ tutorial video, all modules will be immediately accessible. Second, engagement/interactivity through the provision of (i) worksheets, activities, and quizzes with immediate feedback; (ii) a ‘favourites’ and ‘notes’ feature to encourage participants to highlight and reflect on relevant content, a ‘where you left off’ feature to enable participants to immediately return to their last accessed page, and (iii) automated weekly email reminders, provided for the first 6-weeks after enrolling. A booster module is provided 10-weeks after enrolment, providing psychoeducation regarding maintaining wellbeing, a summary/review of the program, and links back to key resources. Materials provided to control participants (outlined below) are integrated within a *Resources* section of the intervention, which also provides links to, and information about, other cancer organisations and websites.

**Table 1:** Finding My Way-Advanced Intervention content

Module	Content and Features
1: Navigating Healthcare	• Covers issues women face when navigating their healthcare, including who is in their treatment team, communication, and making treatment decisions • Activities: Assertive Communication; Decision Making
2: The Unique Challenges	• Covers coping with fear of progression, living with uncertainty, and living well • Activities: Values clarification; Goal setting; Mindfulness; Worry postponement; Therapeutic Writing; Distress Tolerance
3: Physical Symptoms	• Covers common cancer-related and treatment side effects that women may experience, and how they can cope with these • Activities: Fatigue and Pain Activity Pacing; Relaxation
4: Emotional Distress	• Covers common emotional reactions, including anxiety, depression, stress, and anger, and ways to cope with these emotions. • Activities: Behaviour Activation; Cognitive Restructuring; Mindfulness; Therapeutic Writing
5: How You See Yourself	• Covers changes to body image, intimacy, and identity • Activities: Reducing body dissatisfaction; Acceptance; Exploring Intimacy; Core Attributes Exploration
6: Your Family and Friends	• Covers common concerns women have for their family and friends, and coping with social changes that may occur • Activities: Needs exploration; Assertive Communication; Social Support Exploration

#### Control: Breast Cancer Network Australia’s *My Journey* online

Consistent with other web-intervention trials in cancer [24], the control arm will receive a minimal intervention attention-control online. This will comprise a FMW-A control homepage with two picture-tile links to (i) BCNA’s “*My Journey*” online and app resource, and (ii) *Resources* that provide links to information from other cancer organisations and websites. *My Journey* covers all stages of breast cancer, but is tailored to the cancer stage at sign up, such that control participants will receive MBC-relevant content only. *My Journey* for MBC incorporates and extends the gold-standard “Hope & Hurdles” resources previously made available to women upon diagnosis with MBC in Australia. It provides comprehensive information about diagnosis, information specific to metastatic sites, hormone and HER-2 positive MBC, treatments, support options, practical issues, planning ahead, information for loved ones, podcasts, and personal video stories of women with MBC. If FMW-A efficacy is demonstrated, control participants will be given first access to the program as part of the planned post-trial public dissemination.

### Study procedures and timeline

**Fig. 2:**
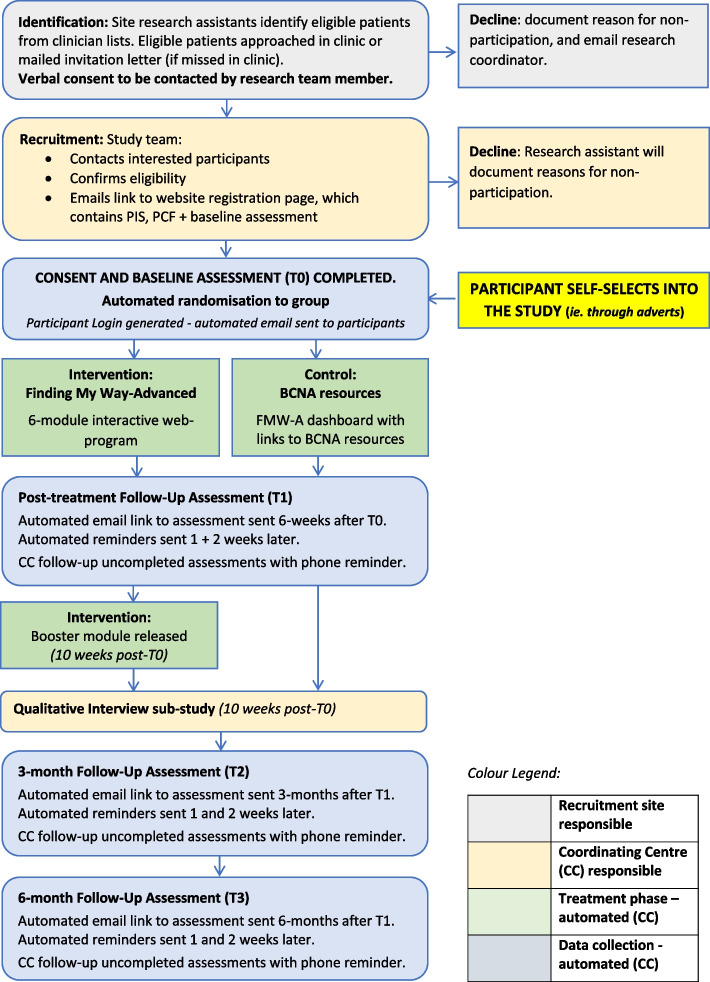
Flow chart of participants through the FMW-A study

Figure 2 outlines the flow of participants through the study. Women eligible for the RCT will be directed to the web-program to register, consent and complete T0 baseline measures for the study, following procedures successfully utilised in our previous studies [27]. Randomisation will then occur with participants immediately redirected to their respective homepages. Following baseline assessment, randomisation will occur via an automated computer-program, with participants allocated to either the Intervention condition, or Minimal Intervention Attention-Control condition. Six weeks after enrolment, participants will receive email notification to complete the T1 post-intervention assessment, with T2 and T3 follow-up questionnaire email notifications sent 3 and 6 months later. Non-responders will receive two automated email reminders, and one telephone reminder to complete follow-up assessments.

### Qualitative examination of user satisfaction

A subset of participants from intervention and control groups will be contacted by telephone at 10-weeks post baseline to schedule a telephone interview for the purposes of obtaining qualitative feedback on user satisfaction with the intervention and/or resources offered as the control condition. Approximately 20 participants will be approached in order of enrolment until saturation of themes occurs. Consenting participants will then be sent a topic guide via email 2 weeks prior to their scheduled interview. Telephone interviews will be conducted and audio recorded, and participants can review their transcripts if requested.

### Timeline

This study is planned to occur over 3 years, with a recruitment window of 18 months to recruit the full sample of 370 participants. The proposed timeline includes 20% of the sample being recruited in Year 1 (2022); 60% in Year 2 (2023); and the final 20% in Year 3 (2024), with the remainder of Year 3 devoted to collection of follow-up data and analysis.

### Measures

**Fig. 3:**
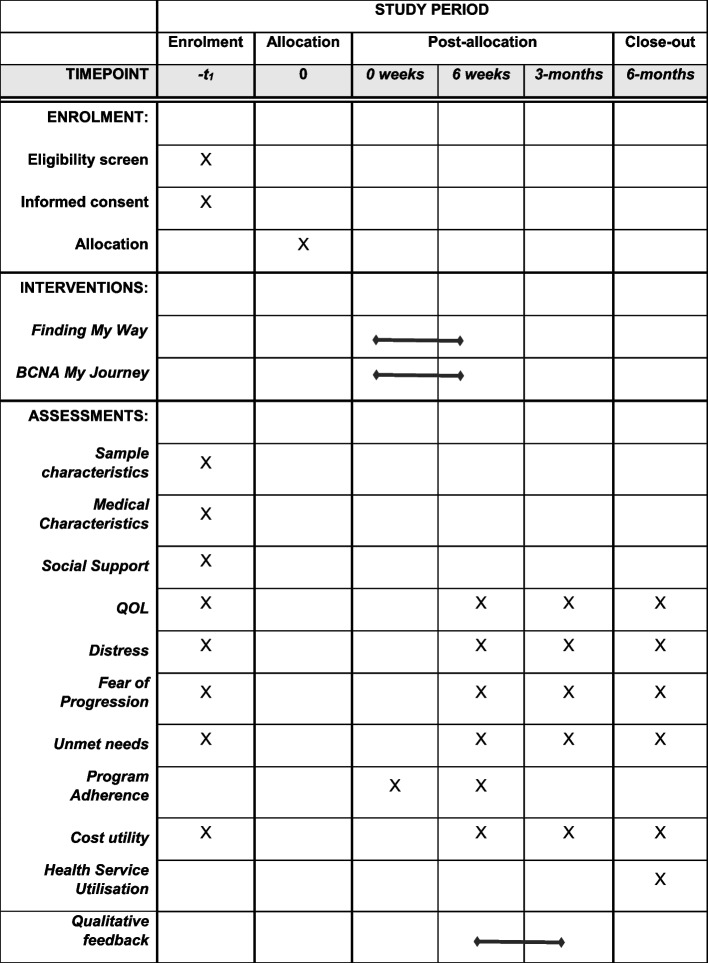
Schedule of enrolment, interventions, and assessments

Table 2 outlines the efficacy outcomes that will be assessed using a battery of validated self-report measures at baseline, post-intervention (primary endpoint), 3-month and 6-month follow-ups, supplemented with website usage data and patient health service use data obtained via data linkage of government databases (‘Services Australia’). Figure 3 outlines the schedule of assessments.

#### Sample characteristics

The demographic, clinical and treatment characteristics will be captured via self-report and confirmed via chart review (where available), and are outlined in Table 2.

#### Primary outcome: mental QOL

Mental QOL will be measured using the EORTC QLQ C-30 emotional functioning subscale [33]. Total scores range from 0 to 100, with higher scores indicating better functioning. QOL has been selected as the primary outcome as it is one of two priority outcomes for MBC nominated by Cancer Australia [2], and has not had sufficient focus in earlier trials [34]. Furthermore, it is a meaningful outcome to patients [2], correlates well with other clinical outcomes and is demonstrated to be sensitive to change [35, 36].

#### Secondary outcomes

Secondary outcome measures assessing QOL, psychosocial and health-economic outcomes, and covariates are summarised in Table 2.

#### User satisfaction

User satisfaction will be assessed using single Likert-style items in the follow-up questionnaire asking participants how helpful they found aspects of the program, and how confident they felt they could apply the program to their everyday life, and through qualitative interviews.

**Table 2:** Measures of Secondary Outcomes, Sample Characteristics, and Baseline Covariates

Domain/Measure	Description
Secondary Outcomes	
QOL	Other dimensions of quality of life will be measured by the EORTC QLQ-C30 subscales (global QOL; physical, social, role, cognitive functioning; physical symptoms) [33].
Distress	The 21-item Depression Anxiety Stress Scale [37]. Assesses anxiety, depression and stress over the previous week; total distress score will be used. The total scale score of the 17-item Post-traumatic Stress Scale-Self-Report (PSS-SR) will be used to measure cancer-specific distress [38]. Items measure symptoms experienced over the past week, rated on a 4-point scale (0 = not at all to 4 = almost always), and anchored to cancer diagnosis as the stressor. Higher scores indicate higher cancer-specific distress
Fear of Progression	The English-version of the 12-item Fear of Progression Questionnaire short form measures levels of anxiety regarding cancer progressing on a five-point Likert scale from 1 (‘never’) to 5 (‘very often’) [39].
Unmet needs	34-item Supportive Care Needs Survey – Short Form [40]. Measures perceived psychosocial, information, physical/activity of daily living, patient care and sexuality needs.
Cost utility	The EuroQol 5 dimensions 5 level (EQ-5D-5L) is a generic preference-based measure [41]. Based on Australian-specific algorithms ranging from − 0.281 to 1, utility scores will be converted into quality adjusted life years (QALYs) required for cost-utility analysis.
Participant Characteristics and Covariates (collected at baseline only)	
Socio-demographic characteristics	Age, marital status, sexual orientation, children, occupational status, annual gross income, educational attainment level, area of residence/postcode, ethnicity.
Medical characteristics (To be assessed via self-report and confirmed via hospital chart review where available)	Date of MBC diagnosis, date of previous BC diagnosis (if applicable), tumour type (hormone status, site of progression), treatments received or planned (surgery, chemotherapy, radiotherapy, hormonal therapy, other), other health conditions, number of prescription medications (to calculate comorbidity index: Rx-Risk score [42], and other support-services accessed (including psychology).
Social support	The Medical Outcome Study Social Support Survey yields a total-scale score and four subscales: emotional/informational, tangible, affectionate, and positive social interactions [43].
Objective Utilisation Data: Website Adherence and Health Service Utilisation	
Intervention adherence	Number of visits, session duration, number of modules completed, pages viewed, and worksheets completed [35].
Services Australia-reported health service use and costs	Medicare Benefits Schedule (MBS) data will be accessed to determine the number and costs of primary care visits, medical consultations, treatments, investigations, and out-of-hospital visits; Pharmaceutical Benefits Scheme (PBS) data to estimate quantity and costs of pharmaceuticals.
Public-hospital costing data (For South Australian recruitment site only)	Centralised costing data in public hospitals (Australian Refined Diagnosis Related Groups (AR-DRGs) will be accessed for determining the number and costs of public hospital inpatient episodes. While this only covers public health sector costs and is not available for participants who self-refer this limitation will apply equally to both groups, due to randomisation.

### Trial oversight

The trial has four levels of oversight. Routine *operational oversight* of the trial is managed by the Coordinating Centre, comprised of principle coordinating investigators (LB, EK, BKo) and affiliated students working on the project. Bi-monthly Project Management Group meetings, comprised of coordinating centre members and three named consumer investigators, oversee *governance and recruitment* aspects of the trial. The Steering Committee, comprised of all investigators, associate investigators, consumer representatives and site coordinators, meet bi-annually at a minimum to provide *strategic oversight* and broader project management and planning. Finally, *data management* for the trial is managed by the Data Management Team, comprised of coordinating centre members, biostatistician (RW) and health economist (BKa). A separate data monitoring committee is not required, due to the automated and self-report nature of the data. Trial conduct will not be externally audited; the trial oversight committees will conduct regular internal audits.

### Safety reporting

Participants will be closely monitored throughout the study by the Coordinating Centre through questionnaires and feedback comments delivered via the website. Participants are free to withdraw from the trial at any time. Reasons for withdrawal will be documented (where provided), and withdrawing participants will still be offered the opportunity to participate in a qualitative interview substudy. Significant adverse effects (e.g., emotional, psychological, and physical) will be monitored and reported to the Southern Adelaide Clinical Human Research Ethics Committee (SAC-HREC).

### Data management

Only Coordinating Centre investigators and web developers will have administrative access to the website and data. All data will be kept in a re-identifiable form using a patient identification code. Electronic data will be stored on secure Flinders University password-protected servers. Data will be used for research publications in academic journals and presentation at scientific meetings and conferences. No data linkage or secondary use of data is planned or anticipated.

### Statistical methods

#### Sample size and justification

The sample size calculation is based on a repeated measures ANCOVA approach, which takes into account the within-subject correlation across measures, using STATA’s “sampsi” routine and our primary outcome of MQOL. Assuming a between-subject SD of 20 points, 1 baseline measure, and 3 post-baseline measurements per subject (post-treatment, 3-months and 6-months) and a within-subject QOL rho = 0.6, *n* = 147 subjects per group will provide 80% power to detect a clinically meaningful difference in MQOL between treatments of 4 points. Based on attrition rates observed in low-intensity MBC interventions (range 4-20%) and our pilot data to date, we will recruit and enrol 185 subjects per group (total *n* = 370; 46 per recruitment site) to allow for 20% attrition. Based on our pilot data where 71% of eligible women consented, 530 women will be approached (equating to approximately 66 *approached* per recruitment site over the 18-month recruitment window, or 3 to 4 per month), to reach the final sample size of 370.

### Primary analyses

Linear Mixed Models (LMM) will be employed following completion of data collection to examine the efficacy of the intervention, in line with the statistical methodology utilised in previous trials our group has published [27, 28]. Analysis will be by intention to treat, defined as all patients who register. LMM will be used to model all continuous outcomes while accounting for covariance between repeated measures on patients and adjusting for baseline measures, which improves precision of estimation of effects. These models will allow for comparing patterns of change over time by testing group by time interaction, and estimating and testing differences between groups at time points of interest via linear contrasts. Mixed models are valid for data that are missing completely at random and missing at random; patterns of missing data will be considered to assess potential missing data mechanisms. In the present study, the fixed main effects will be treatment condition (intervention, control), and time (post-treatment, 3-month, 6 month), with the baseline measure of the outcome entered as a covariate. A random subject effect will be entered into the model to allow the trajectory of each individual to differ by a constant elevation from the group trajectory. Between-group effect sizes and reliable change indices will be calculated to provide a measure of the magnitude and clinical significance of changes, respectively. No interim analysis is planned.

### Secondary analyses

#### Health economics

Within-trial incremental costs associated with the intervention when compared to the control will be estimated using Medicare Benefits Schedule (MBS), Pharmaceutical Benefits Scheme (PBS) and Australian Refined Diagnosis Related Groups (AR-DRG) data. Costs related to the intervention itself will be determined from administrative data collected as part of the trial. The primary health economics outcome will be the incremental cost per unit change in the EORTC QLQ-C30 mental functioning subscale, while the secondary outcome will be the incremental cost per quality adjusted life year (QALY) gained. Within-trial cost-effectiveness with respect to both outcomes will be analysed, allowing for bivariate uncertainty with bootstrapping of participant costs and effects to maintain the covariance structure. To estimate QALY gains, participants will complete the EQ-5D-5L instrument at each assessment. Patient-level measures of utility derived from the EQ-5D-5L instrument will be integrated with survival curves using the quality-adjusted survival analysis (QASA) method. This analysis will include cost-effectiveness, acceptability, net benefit and expected net loss curves to inform decision makers of the optimal strategy at any given threshold for different patient subgroups. Sensitivity analysis will include estimating the incremental cost per unit change in the number of practitioners seen in the previous 3 months based on health services data (via Services Australia data linkage). Of note, given that FMW-A encourages help-seeking, increases in health service utilisation may be observed in a subset of participants; this will be explored qualitatively.

#### Qualitative interview data

Qualitative interview data will be analysed using thematic analysis to identify salient themes in participants’ experience of using the web-based intervention.

### Compliance with regulatory guidelines

The study will be conducted in full conformance with principles of the “Declaration of Helsinki”, Good Clinical Practice (GCP), the Australian National Statement on Ethical Conduct in Human Research (NHMRC, 2007), Australian Code for the Responsible Conduct of Research (2007) and with the laws and regulations of Australia. All study documentation has received ethical approval from the Southern Adelaide Clinical Human Research Ethics Committee (SAC-HREC 2021/HRE00407). Site Specific Assessment (SSA) approval will be sought and obtained from local research governance offices of all participating sites. During the study, the CIA will submit documents recording any serious adverse events or any amendments to this protocol to SAC-HREC. At the conclusion of the study, the CIA will notify SAC-HREC that the study has ended by submitting a final report.

## Discussion

Digital health tools offer one means of addressing the significant access barriers experienced by women with metastatic breast cancer, where symptom burden or physical incapacity, social or geographic isolation, or financial strain preclude participation in face-to-face therapy. However, no RCT has been published to date on the efficacy of a digital health program for this population. While digital health solutions have proliferated in the general community, none have been specifically developed for women with MBC, thus lack the required essential evidence of efficacy, safety, acceptability, accessibility, and content-relevance for this vulnerable population [31]; RCTs of population specific interventions are, therefore, critical. To our knowledge, *Finding My Way-Advanced* is unique in addressing this research and resource gap.

The large-scale nature of this national multi-site RCT will enable examination of the *efficacy* of the program in improving mental and overall quality of life, reducing distress including fear of progression, unmet needs, and health service use. Furthermore, this study offers the opportunity to evaluate potential *moderators* of change and determine how they overlap or differ to those identified in our earlier *Finding My Way* program for those treated with curative intent [44]. This information has clinical utility in aiding healthcare professionals in identifying for whom and under what conditions digital health is beneficial, and who may be better suited to other modalities.

If the intervention efficacy is evidenced, this program has individual, system-level, national and international significance. For individuals, the program provides an accessible and cost-effective method of obtaining therapeutic benefits. By improving mental QOL and distress, the overall burden of their disease is reduced via potentially reducing their severity of side effects/toxicities [6, 7], and increase ability to tolerate treatments [8], while increasing capacity for valued activities. This in turn has the potential for system-level benefits, via reduced health service utilisation / health system burden [9].

Nationally, the already developed and approved implementation and dissemination plan will ensure immediate and ongoing population-level scaling of *Finding My Way-Advanced* in Australia via the national body, BCNA. There is also strong potential for international scaling, as demonstrated by our group’s prior history of trialling adaptations of the original *Finding My Way* digital health program in the USA [45] and the UK [46], and a commitment to conduct a replication study of FMW-A in the UK is already in place, funding-dependent. This program is readily and directly scalable to the English-speaking world, and can form a foundation for culturally-appropriate adaptations and translation to other languages or settings. Finally, FMW-A provides a template for delivering psychological treatment for individuals diagnosed with other metastatic cancers, or chronic health conditions that face similar physical incapacity, social isolation and/or financial strains and access barriers.

In summary, this study is significant in providing an innovative method for providing psychological treatment for a high-need but underserved population, while evaluating the benefits to the health-system and patients. It brings strong methodological rigour to the research design, will provide important contributions to the digital health evidence-base and, most importantly, addresses the recognised paucity of psychological interventions developed specifically for women with MBC.

## Supplementary Information

**Additional file 1.**

## Data Availability

The datasets generated during and/or analysed during the current study are available from the corresponding author on reasonable request. We cannot obtain ethical approval to place clinical trial data on a public repository, due to the highly confidential / sensitive nature of the data, which participants have not consented to be released.

## Abbreviations

ACT: Australian Capital Territory
ANCOVA: Analysis of Covariance
AR-DRGs: Australian Refined Diagnosis Related Groups
BCNA: Breast Cancer Network Australia
CBT: cognitive behaviour therapy
FMW-A: Finding My Way – Advanced
ID code: identification code
LMM: linear mixed models
MBC: metastatic breast cancer
MBS: Medicare Benefits Schedule
MQOL: mental quality of life
NSW: New South Wales
PBS: Pharmaceutical Benefits Scheme
QALY: quality adjusted life years
QOL: quality of life
RCT: randomised controlled trial
SAC-HREC: Southern Adelaide Clinical Human Research Ethics Committee
